# Effects of fecal microbiota transplantation and probiotics on the gut microbiome in antibiotic-treated septic patients: A pilot randomized controlled trial

**DOI:** 10.1080/21505594.2026.2668764

**Published:** 2026-05-22

**Authors:** Yuanzhuo Chen, Jian Zhao, Jiangman Zhao, Qiyi Chen, Shi Dong, Shaohua Jia, Yi Zhao, Di Hao, Yangyang Yin, Sitong Lin, Yanqing Chen, Yugang Zhuang, Hu Peng

**Affiliations:** a Department of Emergency, Shanghai Tenth People’s Hospital, School of Medicine, Tongji University, Shanghai, China; b Department of Medicine, Shanghai Biotecan Pharmaceuticals Co., Ltd., Shanghai, China; c Department of Medicine, Zhangjiang Institute of Medical Innovation, Shanghai, China; d Department of Colorectal Disease, Intestinal Microenvironment Treatment Center, Shanghai Tenth People’s Hospital, Tongji University School of Medicine, Shanghai, China; e Institute of Gut Microbiota Research and Engineering Development, Shanghai Tenth People’s Hospital, Tongji University School of Medicine, Shanghai, China; f Department of Critical Care Medicine, Shanghai Tenth People’s Hospital, School of Medicine, Tongji University, Shanghai, China; g Department of Key Laboratory of Pathogen-Host Interaction, Ministry of Education, Tongji University, Shanghai, China

**Keywords:** Sepsis, fecal microbiota transplantation, gut microbiota, broad-spectrum antibiotics, probiotics

## Abstract

Broad-spectrum antibiotics, essential for sepsis management in critically ill patients, cause significant gut dysbiosis. Restoring gut microbiota may improve outcomes, but the efficacy of interventions like fecal microbiota transplantation (FMT) and probiotics in this setting remains underexplored. This study aims to evaluate the feasibility and potential efficacy of FMT versus probiotics on gut microbiome restoration and inflammatory markers in critically ill, antibiotic-treated sepsis patients. In this single-center, prospective, exploratory pilot RCT, 40 sepsis patients were were randomized 2:1:1 to: Control (*n* = 20, antibiotics treatment), Probiotics (*n* = 10, antibiotics treatment combined one week of probiotics), and FMT (*n* = 10, antibiotics treatment combined one week of FMT) groups. Gut microbiota composition was analyzed using 16S rDNA sequencing, and clinical inflammatory markers were assessed at baseline, one week, and two weeks post-treatment. FMT significantly mitigated antibiotic-induced reductions in microbial diversity. At 2 weeks, the FMT group exhibited higher alpha-diversity (Chao1 index, *p* = 0.0125; Shannon/Simpson trends *p* = 0.06) compared to Control and Probiotics groups. FMT increased beneficial *Bacteroides* abundance and reduced *Enterobacteriaceae*. BugBase analysis revealed FMT significantly lowered pathogenic potential of gut microbiota (*p* = 0.021). Donor-recipient analysis showed FMT shifted recipient microbiomes toward donor enterotype. This study provides preliminary evidence that FMT, but not the probiotic regimen, effectively restores gut microbiome diversity and composition, reduces pathogenic potential, and may improve clinical outcomes in critically ill sepsis patients after broad-spectrum antibiotics. This study was registered on ClinicalTrials.gov (NCT05578196).

## Introduction

Sepsis represents a critical global public health challenge, particularly in intensive care units (ICUs), where it substantially contributes to mortality [1, 2]. Although antibiotics are essential for controlling infection, they disrupt gut microbial diversity, leading to gut dysbiosis, increased colonization by opportunistic pathogens, intestinal barrier impairment, and exacerbation of systemic inflammation [3]. For example, the gut microbiota has been reported to play a protective role in the host defense against pneumococcal pneumonia [4]. Multiple studies have demonstrated that the use of anti-anaerobic antibiotics leads to the loss of beneficial anaerobic gut microbiota, which adversely affects systemic immune responses and correlates with higher mortality in sepsis patients [5, 6].

Recent studies have focused on microbiome-restorative strategies to counteract antibiotic-induced dysbiosis and improve sepsis outcomes in ICU settings. Selective decontamination of the digestive tract (SDD) did not lead to a significant reduction in antibiotic resistance [7], and its effect on in-hospital mortality was not confirmed in a large randomized clinical trial [8]. Meta-analyses showed synbiotics was a safe and effective nutritional intervention for reducing septic complications in critically ill patients, while probiotic therapy alone has not demonstrated a significant advantage over placebo [9]. It should be noted that probiotic formulations vary widely in composition (strain types, dosages, delivery methods), and their effects are likely context-dependent, varying with patient population, clinical setting, and concurrent treatments.

Fecal microbiota transplantation (FMT), a direct microbial restoration strategy, is now guideline-recommended for various gastrointestinal diseases, especially recurrent*Clostridioides difficile* infection [10]. Preclinical data suggest FMT of sepsis can restore microbial diversity, enhance short-chain fatty acid production, and reduce inflammatory cytokines, thereby decreasing mortality in septic mouse models [11]. A case reported FMT use in critically ill patients to eradicate multidrug-resistant bacterial colonization or refractory infections [12]. Nevertheless, most evidence arises from mouse models or case report, and robust randomized trials in sepsis remain scarce.

Previous studies have established FMT’s safety and efficacy profile in various gastrointestinal diseases [13]. While, its application in sepsis remains limited, with safety concerns in critically ill patients persisting. This single-center, exploratory pilot study aims to evaluate the safety, feasibility, microbiome recovery, and inflammatory outcomes of FMT compared to probiotics in critically ill, antibiotic-treated sepsis patients.

## Methods

### Study participants

This is a single-center, prospective, exploratory randomized controlled pilot trial. Forty patients with sepsis were enrolled in the Emergency Intensive Care Unit (EICU) at Shanghai Tenth People’s Hospital affiliated to Tongji University between Oct 2022 and Sep 2024. Inclusion criteria: age ≥ 14 years, diagnosed with sepsis based on the Third International Consensus Definitions for Sepsis and Septic Shock (Sepsis-3) [14], and SOFA score ≥ 2. Exclusion criteria: advanced malignancy, systemic immunosuppression (defined as receiving immunosuppressive medications, HIV with CD4 < 200 cells/μL, or organ transplantation), pregnancy, severe intestinal ulcers/perforation, or inability to receive treatment orally or via artificial feeding. This study was approved by the ethics committee of Shanghai Tenth People’s Hospital (22k215) and registered on ClinicalTrials.gov (NCT05578196). The study protocol complied with the Declaration of Helsinki. Informed consent was obtained from all participants or their legal representatives.

### Study procedure and randomization

**Figure 1.**
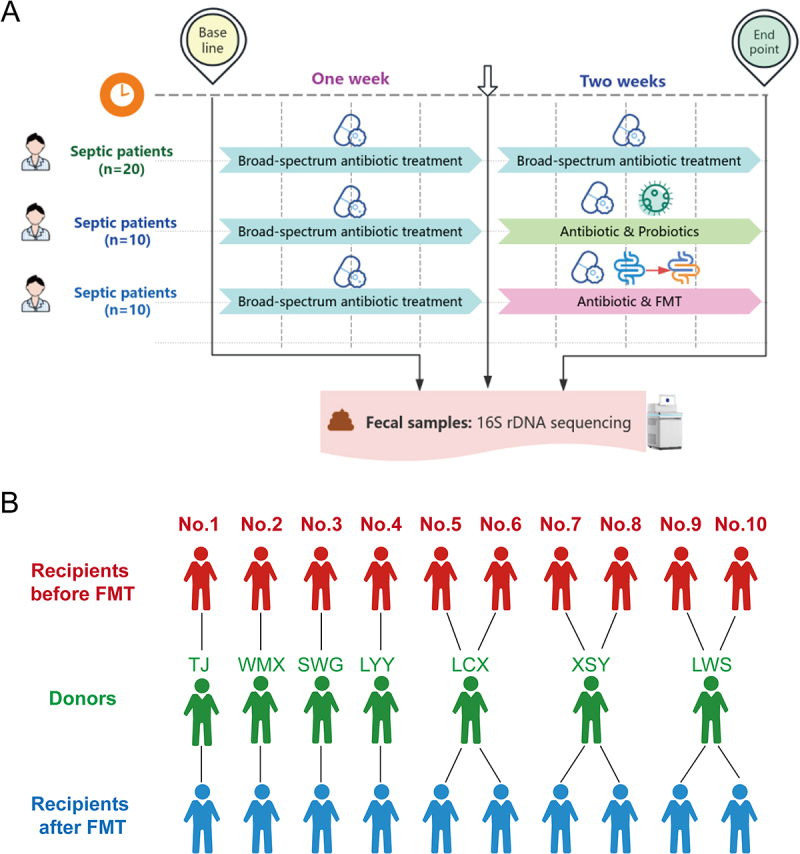
Study design and donor-recipient matching. (a) Flowchart of the pilot randomized controlled trial. Forty septic patients receiving broad-spectrum antibiotics were randomized into three groups: control group (*n* = 20, continued antibiotics), Probiotics group (*n* = 10, continued antibiotics + Bifidobacterium triple viable capsules for 1 week), and fecal microbiota transplantation (FMT) group (*n* = 10, continued antibiotics +6-day FMT regimen via nasoenteric tube). Fecal samples for 16S rDNA sequencing were collected from all participants at baseline, Week 1, and Week 2 (study endpoint). (B) FMT donor-recipient matching matrix. The diagram illustrates the allocation of fecal microbiota suspensions from the seven healthy donors to the ten recipients in the FMT group.

Forty participants were randomized into three groups using a computer-generated randomization sequence: Control (*n* = 20), Probiotics (*n* = 10), and FMT (*n* = 10) groups, as illustrated in Figure 1 (A–B). The 2:1:1 randomization ratio was chosen to balance baseline microbiome characterization with intervention assessment in this exploratory pilot study. Due to the nature of interventions, blinding of patients and care providers was not feasible.

All groups received broad-spectrum antibiotics, according to clinical requirements. Antibiotic exposure (drug class and duration) was illustrated in Supplementary Figure S1, during hospitalization.

After one week, interventions were administered:

1. Control group: Continued antibiotics and 100 ml of normal saline via nasoenteric tube. The 100 mL normal saline administered to the control group served to control for the background effects of the vehicle solution used in FMT (to exclude any potential influence of saline infusion itself).
2. Probiotics group: Continued antibiotics and Peifeikang probiotics (*Bifidobacterium* triple viable powder, 210 mg per capsule) twice daily, 8 capsules/day for one week. Peifeikang (*Bifidobacterium* triple viable powder) was administered concurrently with antibiotics to prevent antibiotic-associated diarrhea and mitigate gut dysbiosis. This formulation, containing *Bifidobacterium longum, Lactobacillus acidophilus* and *Enterococcus faecalis* species, is widely used in China and supported by expert consensus and multiple studies demonstrating its safety and clinical benefit in antibiotic-treated or critically ill patients [15–17].
3. FMT Group: Continued antibiotics and 100 ml fecal microbiota solution via nasoenteric tube daily for six days.

Stool and blood samples were collected at baseline, week 1, and week 2 for 16S rDNA sequencing and analysis of inflammatory markers. No patients were lost to follow-up during the two-week intervention period and 28-day interview.

### Healthy donor screening, fecal microbiota suspension processing, and FMT procedure

Healthy donors were screened according to the Chinese expert consensus on screening and management of fecal microbiota transplantation donors [18]. The standard donor screening criteria cover 6 dimensions, including physiology, psychology, personal history, the stability, persistence, and tolerance to dietary restriction, which minimize risks of disease transmissions. In this study, 7 donors were enrolled. A total of 10 fresh feces were donated by 7 donors, producing 10 batches of the fecal bacterial suspension. Quality control of the donated feces, processed suspension, and FMT management referenced the Expert consensus on clinical application management of fecal microbiota transplantation [19]. To ensure biosafety and consistency, strict batch-to-batch quality control was implemented. After each batch was prepared, random aliquots were collected for pathogen and quality testing. All suspensions were screened negative for *Clostridium difficile, Bacterium flexuosus, Salmonella, Shigella, Shiga-toxin-producing escherichia coli, Norwalk viruses, rotavirus, SARS-CoV-2*, and common pathogens. In addition, multi-drug resistance genes, including extended-spectrum β-lactamases and carbapenemases, were tested and confirmed negative. The viability of bacterial cells in the suspension was maintained at ≥80%, and the viable bacterial count exceeded 5 × 10^8^ cells/mL. Each batch underwent 16S rDNA sequencing to assess microbial composition and confirm consistency. For FMT administration, the fecal bacterial suspension was packaged in 50 mL tubes, and each patient received 2 tubes (100 mL total) once daily via a nasoduodenal tube over approximately 10 minutes. This procedure was repeated for six consecutive days, resulting in a total administered volume of 600 mL per patient (cumulative viable bacterial dose: approximately 3 × 10^11^ cells per patient).

### Fecal DNA extraction and 16S rDNA sequencing

Fecal DNA was extracted using the PowerFecal™ DNA isolation kit (MoBio Laboratories), following manufacturer’s protocol. DNA concentration and purity were assessed using a NanoDrop ND-1000 spectrophotometer (Thermo Fisher Scientific) and agarose gel electrophoresis. The V4 region of the bacterial 16S rRNA gene was amplified via polymerase chain reaction (PCR) using the primers 515F (5’-GTGCCAGCMGCCGCGGTAA-3’) and 806 R (5’-GGACTACHVGGGTWTCTAAT-3’). Resulting amplicons were purified using Agencourt AMPure XP Beads (Beckman Coulter, Indianapolis, IN) and quantified with the PicoGreen dsDNA Assay Kit (Invitrogen, Carlsbad, CA, USA). Equimolar amounts of purified amplicons were pooled and subjected to paired-end sequencing (2 × 150 bp) on the Illumina NovaSeq 6000 platform.

### Bioinformatics

Raw sequencing reads were demultiplexed based on exact barcode matches. Subsequent quality filtering removed sequences that were: (i) shorter than 150 bp, (ii) possessed an average Phred quality score below 20, (iii) contained ambiguous base calls (N’s), or (iv) contained mononucleotide repeats exceeding 8 bp in length. Paired-end reads were merged using Vsearch v2.22.1. Amplicon Sequence Variants (ASVs) were generated via: dereplication, UNOISE2 denoising, de novo chimera removal, and clustering at 100% similarity. ASVs were taxonomically classified against the SILVA 138 database using QIIME2. Sample sequencing depth was normalized by proportional scaling. Alpha diversity (Shannon, Simpson, Chao1) and beta diversity were calculated in QIIME2. Group differences in alpha diversity were assessed by Kruskal–Wallis tests, followed by post-hoc Dunn’s test for pairwise comparisons. Differential abundance analysis across taxonomic ranks employed Kruskal-Wallis/Wilcoxon tests and metagenomeSeq package. Linear discriminant analysis effect size (LEfSe) was performed to identify differentially abundant taxa across groups (LDA > 2.0, *p* < 0.05). Microbial functions were predicted by PICRUSt 2 and BugBase based on high-quality sequences. The statistical significance of differences in the predicted Kyoto Encyclopedia of Genes and Genomes (KEGG) pathway abundances (from PICRUSt2) and potentially pathogenic (from BugBase) among groups was assessed in STAMP v2.1.3 using Kruskal-Wallis tests with post-hoc Dunn’s tests for multi-group comparisons, and Wilcoxon rank-sum tests for two-group comparisons. Benjamini–Hochberg false discovery rate (FDR) correction was applied to correct for multiple comparisons where appropriate. Enterotype classification was performed via partitioning around medoids (PAM) clustering. Cluster stability was optimized using the Calinski-Harabasz criterion to determine the most appropriate number of enterotypes [20].

To evaluate donor microbial engraftment in recipients following FMT, we quantified the Colonizer-to-Resident Ratio (C2R) for each patient, which was defined as the quotient of colonizer abundance over resident abundance [21]. We also computed Bray-Curtis dissimilarities across all samples and visualized community structures via principal coordinates analysis (PCoA). We further quantified donor influence by examining the association between clinical outcomes and the Bray-Curtis distance linking post-FMT recipients to their respective donors.

### Safety and outcome assessment

Patients were closely monitored for safety throughout the initiation of the intervention until hospital discharge. Monitoring included daily clinical assessments for new-onset fever, diarrhea, abdominal distension and pain, vomiting, or other symptoms. Vital signs were tracked continuously as part of standard ICU care. Any adverse events were to be recorded, assessed for relatedness to the study intervention. The primary outcome was the alteration of gut microbiota composition, assessed through 16S rDNA sequencing. Secondary outcomes included inflammatory markers – white blood cell (WBC) count and C-reactive protein (CRP) levels as well as 28-day mortality and length of stay in the intensive care unit (ICU). Additional data collected encompassed patient demographics and baseline characteristics (age, sex, body mass index [BMI], and comorbidities), Sequential Organ Failure Assessment (SOFA) scores, immunological parameters (including interleukin [IL]-6, IL-8, IL-10, T-cell subsets, B cells, and natural killer [NK] cells), sites of infection, pathogen profiles, and antibiotic usage.

### Statistical analysis

In this study, continuous variables were expressed as mean±standard deviation, and categorical variables as frequencies and percentages. Baseline categorical variables were compared using chi-square or Fisher’s exact tests, while continuous variables were compared with one-way ANOVA or Kruskal–Wallis tests. Clinical outcomes, such as changes in white blood cell count and C-reactive protein levels, were analyzed via repeated measures ANOVA. All tests were two-sided, with statistical significance set at *p* < 0.05.

## Results

### Baseline characteristics, safety and clinical outcomes

Baseline characteristics were comparable across Control (*n* = 20), Probiotics (*n* = 10), and FMT (*n* = 10) groups for demographics, comorbidities, and most laboratory parameters (*p* > 0.05; Table 1, Supplementary Table S1). However, baseline imbalances existed: significant intergroup differences existed for SOFA scores (*p* = 0.042), IL-8 levels (*p* = 0.003), age (*p* = 0.060), and β-lactam antibiotic exposure (*p* = 0.037). The 28-day mortality rate was 25.0% (5/20) in Control group, 20.0% (2/10) in Probiotics groups, and 0% (0/10) in the FMT group (*p* = 0.297). This exploratory finding should be interpreted cautiously given the baseline imbalances and small sample size. ICU length of stay did not differ significantly (*p* = 0.407). Although WBC and CRP levels decreased post-treatment across all groups-with the most pronounced WBC reduction in FMT-no statistically significant intergroup differences were observed. No adverse events related to FMT or probiotic administration were observed during the study period.

**Table 1.** Baseline characteristics of the study participants.

Characteristics	Control (*n* = 20)	Probiotics (*n* = 10)	FMT (*n* = 10)	*P* value
**Demography**				
Age	68.2 ± 10.4	63.5 ± 20.2	53.2 ± 19.2	0.060
Male	12 (60.0%)	8 (80.0%)	5 (50.0%)	0.363
BMI	21.5 ± 4.0	22.2 ± 4.3	24.4 ± 8.6	0.410
**Disease score**				
SOFA	8.1 ± 3.0	4.9 ± 2.4	8.0 ± 3.8	0.042
**Comorbidity**				
Heart failure	10 (50.0%)	8 (80.0%)	7 (70.0%)	0.237
COPD	5 (25.0%)	3 (30.0%)	3 (30.0%)	0.939
Chronic kidney disease	4 (20.0%)	1 (10.0%)	3 (30.0%)	0.535
Diabetes	12 (60.0%)	6 (60.0%)	4 (40.0%)	0.545
**Laboratory test**				
WBC	10.3 ± 4.5	16.7 ± 15.6	13.8 ± 5.6	0.177
CRP	87.5 (50.4–153.8)	61.6 (27.8–127.4)	99.3 (22.0–182.8)	0.780
IL-6	60.2 (33.1–210.7)	173.2 (88.2–367.2)	57.0 (44.7–152.9)	0.627
IL-8	246.2 (105.5–458.5)	612.6 (197.6–924.0)	50.7 (14.0–99.2)	0.003
IL-10	7.1 (4.4–8.5)	11.9 (5.4–24.8)	14.0 (8.2–23.0)	0.214
TNF	1.9 (1.5–2.9)	7.5 (5.3–10.6)	4.0 (3.7–10.3)	0.231
Total T-cell	759.0 (364.0–999.0)	497.5 (377.2–701.0)	932.0 (603.5–1148.5)	0.139
Th-cell	421.0 (260.5–584.5)	336.0 (170.2–448.5)	566.5 (425.2–677.8)	0.164
Ts/Tc-cell	267.0 (150.0–448.5)	158.0 (111.0–255.8)	295.5 (153.8–357.2)	0.214
B-cell	121.0 (59.5–256.5)	121.0 (56.2–259.2)	103.5 (62.2–161.8)	0.892
NK-cell	148.0 (72.5–263.5)	144.0 (109.2–233.5)	58.0 (50.5–159.5)	0.339
**Infection site**				
Respiratory	19 (95.0%)	9 (90.0%)	7 (70.0%)	0.194
Urinary	1 (5.0%)	1 (10.0%)	2 (20.0%)	0.533
Abdomen	1 (5.0%)	1 (10.0%)	4 (40.0%)	0.075
Blood	4 (20.0%)	3 (30.0%)	3 (30.0%)	0.694
**Microorganisms**				
Bacteria	17 (85.0%)	10 (100.0%)	8 (80.0%)	0.573
Fungi	7 (35.0%)	0 (0)	4 (40.0%)	0.082
Virus	1 (5.0%)	0 (0)	1 (10.0%)	0.999
**Antibiotics**				
β-lactams	16 (80.0%)	9 (90.0%)	4 (40.0%)	0.037
Aminoglycosides	0 (0)	0 (0)	6 (60.0%)	0.001
Tetracyclines	7 (35.0%)	1 (10.0%)	0 (0)	0.071
Quinolones	6 (30.0%)	5 (50.0%)	1 (10.0%)	0.170
Macrolides	0 (0)	2 (20.0%)	3 (30.0%)	0.038
Peptides	7 (35.0%)	2 (20.0%)	2 (20.0%)	0.727
Glycopeptides	0 (0)	1 (10.0%)	1 (10.0%)	0.239
SMZ	2 (10.0%)	1 (10.0%)	2 (20.0%)	0.825
antifungal.drugs	8 (40.0%)	2 (20.0%)	3 (30.0%)	0.586
antivirus.drugs	1 (5.0%)	0 (0)	0 (0)	0.999
**Outcome**				
28-day mortality	5 (25.0%)	2 (20.0%)	0 (0)	0.297
Los_ICU	22.2 ± 7.6	25.3 ± 10.2	20.6 ± 5.2	0.407

### Gut microbiome restoration by FMT

#### FMT can restore antibiotic-induced gut microbiome diversity loss

**Figure 2.**
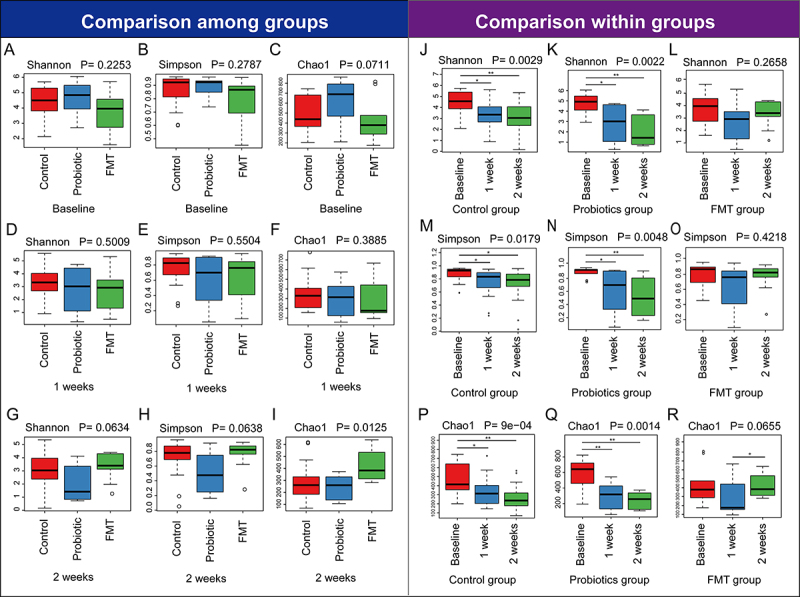
Longitudinal analysis of gut microbiome alpha diversity. Alpha diversity indices were compared across and within the Control, Probiotics, and FMT groups over time. (A–C) Comparisons among the three groups at baseline for the (a) Shannon, (b) Simpson, and (c) Chao1 indices. (D–F) Inter-group comparisons at Week 1. (G–I) Inter-group comparisons at Week 2, showing a significant recovery of Chao1 in the FMT group. (J–L) Longitudinal comparisons of the Shannon index within each group from baseline to Week 2. (M–O) Longitudinal comparisons of the Simpson index within each group. (P–R) Longitudinal comparisons of the Chao1 index within each group, demonstrating a recovery over time in the FMT group.

16S rDNA sequencing analysis showed no significant differences in alpha diversity – measured by Shannon (Figure 2A, 2D), Simpson (Figure 2B, 2E), and Chao1 (Figure 2C, 2F) indices among the Control, Probiotic, and FMT groups, at baseline and one week (Supplementary Table S2). Comparative analysis among groups (Figure 2J-R) revealed significant reductions in Shannon, Simpson, and Chao1 indices at Weeks 1 post-antibiotics in Control, Probiotics groups and FMT group. The alpha diversity of patients in FMT group has obviously recovery after FMT treatment for one week (Figure 2L, O, R). The results demonstrated significantly higher species richness (Chao1 index, *p* = 0.0125, Figure 2I) and trends toward increased Shannon/Simpson diversity (*p* ≈0.06, Figure 2G-H) in the FMT group versus Controls/Probiotics at Week 2. These results suggest that FMT could effectively preserve gut microbiome species richness despite antibiotic exposure, demonstrating potential long-term benefits for critically ill patients.

#### Taxonomic composition shifts

**Figure 3.**
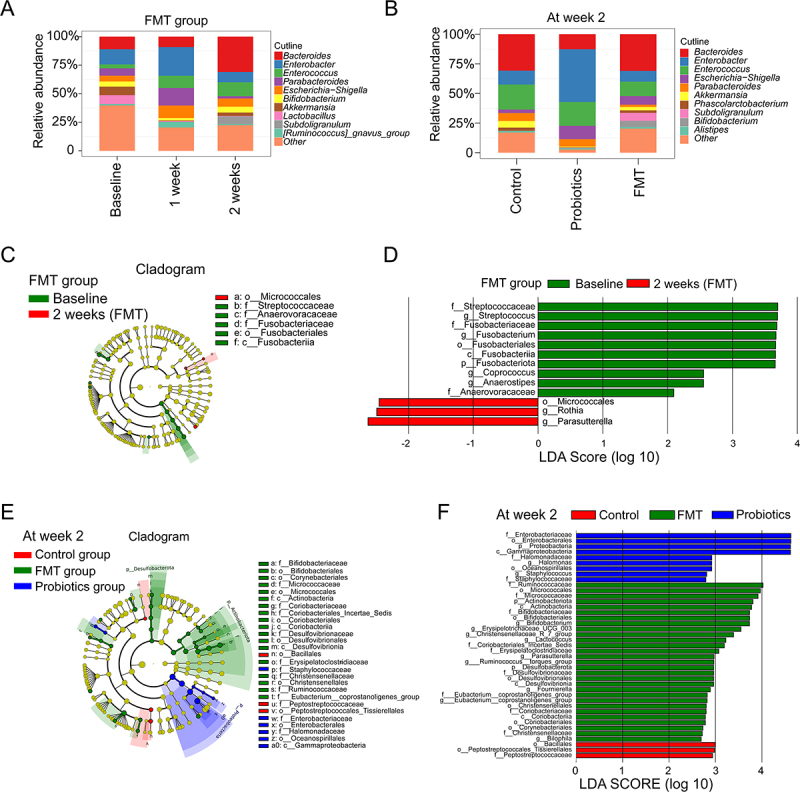
Temporal and comparative shifts in gut microbial composition following intervention. (A) Longitudinal genus-level composition within the FMT group. Stacked bar plots show the relative abundance of the top 10 most abundant genera and “other” taxa in the FMT group at baseline, Week 1 (post-antibiotics), and Week 2 (post-FMT). (B) Comparative genus-level composition across groups at Week 2. Stacked bar plots compare the relative abundance of the top 10 most abundant genera and “Other” taxa in the Control, Probiotics, and FMT groups at the study endpoint (Week 2). (C–D) Differential taxonomic abundance within the FMT group over time. (C) Cladogram from Linear Discriminant Analysis Effect Size (LEfSe) analysis highlighting taxa that were differentially abundant across the three timepoints (Baseline, Week 1, Week 2) specifically within the FMT group (LDA Score > 2.0, *p* < 0.05). (D) The corresponding LDA score bar chart quantifies the effect size of differentially abundant taxa. (E–F) Differential taxonomic abundance across treatment groups at Week 2. (E) Cladogram from LEfSe analysis identifying taxa that were differentially abundant among the Control, Probiotics, and FMT groups at Week 2 (LDA Score > 2.0, *p* < 0.05). (F) The corresponding LDA score bar chart quantifies the effect size of differentially abundant taxa across the three groups.

Longitudinal analysis revealed divergent taxonomic shifts across groups (Supplementary Figure S2, Supplementary Table S3). Control and Probiotics groups exhibited increasing *Enterococcus* and *Enterobacter* abundances alongside declining genus-level diversity over time (Supplementary Figure S2 A-F), FMT recipients demonstrated significant reversal of antibiotic-driven changes – notably increasing *Bacteroides* while reducing *Enterobacter* (Figure 3A-B). *Micrococcales* was significantly enriched after FMT treatment (Figure 3 C-D). By Week 2, intergroup comparisons confirmed distinct compositional profiles (Figure 3E-F): FMT samples maintained the richest microbiota with prominent enrichment of beneficial taxa (*Bifidobacteriaceae*, *Coriobacteriaceae*), contrasting with Probiotics group (dominance of *Enterobacteriaceae*) and Control group (prevalence of *Peptostreptococcaceae*).

#### Predicted functional and pathogenic alterations

**Figure 4.**
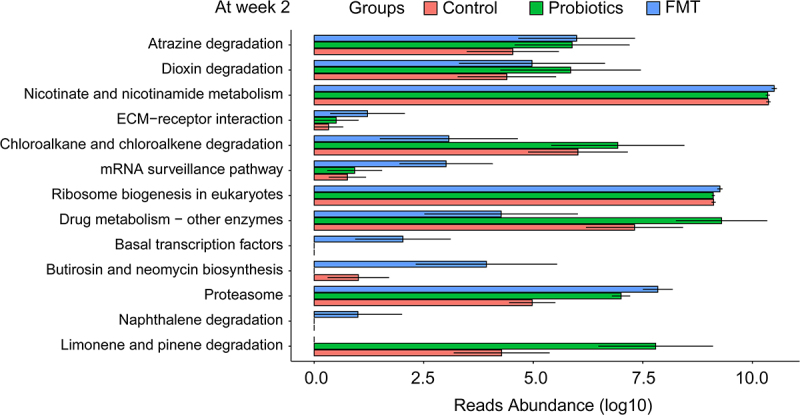
Predicted differential enrichment of microbial metabolic pathways based on KEGG at Week 2 among Control, probiotic and FMT groups by Picrust2. Bar chart depicting the relative abundance (log10-transformed) of select KEGG pathways at the study endpoint (Week 2), as predicted by PICRUSt2 from 16S rRNA gene data. Pathways are shown for the Control, probiotic, and FMT groups.

**Figure 5.**
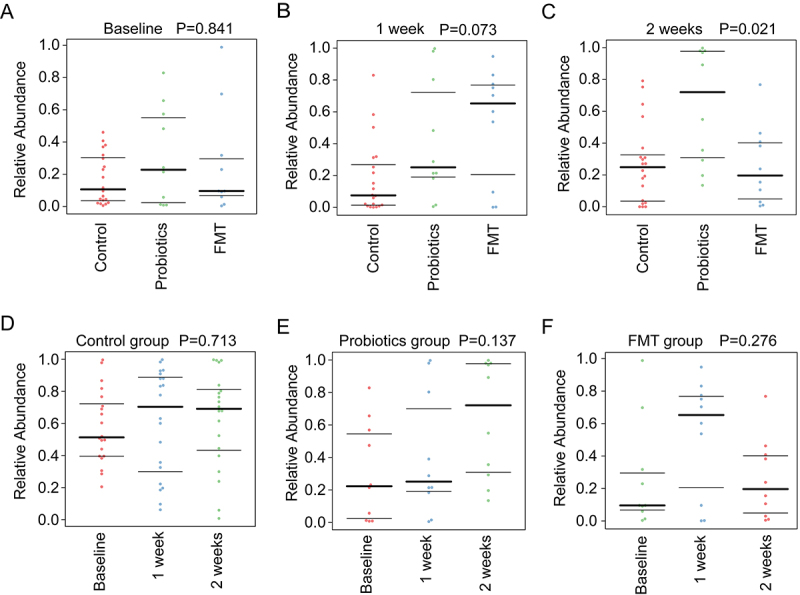
Temporal analysis of the predicted pathogenic potential of the gut microbiota using BugBase from 16S rRNA gene data. (A–C) Comparisons of pathogenic potential among the Control, Probiotics, and FMT groups at (a) Baseline, (b) Week 1, and (c) Week 2. A statistically significant reduction in the FMT group compared to the other groups was observed at the 2-week endpoint. (D–F) Longitudinal analysis of pathogenic potential within each treatment group over time: (d) Control, (e) Probiotics, and (f) FMT. The FMT group demonstrates a visual trend of reduction in pathogenic potential following the intervention.

Functional profiling predicted via PICRUSt2 revealed, at the 2-week endpoint, FMT recipients demonstrated substantially enhanced enrichment in xenobiotic degradation pathways, energy metabolism, and antibiotic resistance modulation compared to Control and Probiotics groups (*p* < 0.05; Figure 4). Pathogenic potential predicted using BugBase further highlighted FMT’s protective role (Figure 5A-F). Pathogen analysis demonstrated significantly lower pathogenic taxa in FMT versus Controls and Probiotics (*p* = 0.021, Figure 5C), correlating inversely with restored *Bacteroides* abundance.

Collectively, these findings demonstrate FMT’s superior efficacy over probiotics in restoring both taxonomic structure and functional capacity of the antibiotic-disrupted gut microbiome (Figure 5A-F). Critically, FMT significantly attenuated the antibiotic-driven expansion of potential pathogens suggesting durable protection against dysbiosis. This pathogen suppression aligns with FMT’s established role in rebuilding microbial diversity and enriching beneficial taxa, highlighting its multifaceted restorative potential.

### Mechanisms underlying FMT efficacy

#### Enterotype-driven microbiome restructuring

Taxonomic profiling revealed a significant shift toward donor-like microbial ecology in FMT recipients. While donors predominantly exhibited the *Bacteroides*-dominant enterotype (6/7), pre-FMT recipients exhibited the *Parabacteroides*-dominant enterotype (5/10, 50%). Post-FMT, 80% (8/10) of recipients achieved accurate enterotype classification, with 60% (6/10) transitioning to the donor-characteristic *Bacteroides* enterotype. This restructuring correlated with increased colonization by donor-derived *Bacteroides* strains, confirming successful engraftment of keystone taxa critical for gut ecosystem stability.

#### Donor-recipient engraftment dynamics

**Figure 6.**
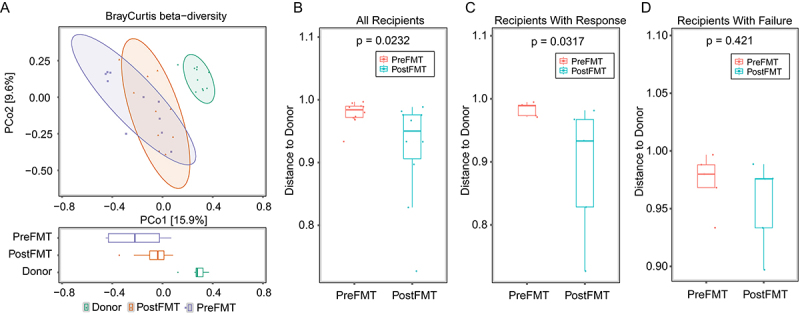
Analysis of donor microbiota engraftment and community convergence in FMT recipients. (a) Principal coordinates analysis (PCoA) plot of Bray-Curtis dissimilarity, illustrating the overall microbial community structure (beta-diversity) among healthy donors, recipients before FMT (pre-FMT), and recipients after FMT (post-FMT). (B-D) Bray-Curtis dissimilarity (BC distance) between each recipient and their respective donor, before (pre-FMT) and after (post-FMT) the intervention. (b) All FMT recipients combined (*n* = 10) showed a significant decrease in distance to their donor (*p* = 0.0232). (c) The subset of recipients classified as “responders” showed a significant convergence toward their donor’s microbiota (*p* = 0.0317). (d) The “failure” group (non-responders) showed no significant change in community distance to their donor post-FMT (*p* = 0.421).

To visually capture the microbial restructuring, we analyzed the top 10 most significantly altered genera. As shown in Supplementary Figure S3, the post-FMT recipient microbiota demonstrated a clear convergence toward the donor profile, with the abundance of these key genera shifting to an intermediate state between the pre-FMT and donor levels. Microbial engraftment efficiency was assessed through Principal Coordinates Analysis (PCoA) plot of Bray-Curtis dissimilarity (Figure 6A). For Colonizer-to-Resident Ratio (C2R), 2 responders (defined by reduced SOFA scores) exhibited positive C2R values (2/10), indicating dominant donor-derived colonization, whereas non-responders showed negligible colonization. For community convergence, Bray-Curtis dissimilarity analysis demonstrated that all FMT recipients combined (*n* = 10) showed a significant decrease in distance to their donor (Figure 6B, *p* = 0.0232). Respectively, post-FMT responders’ microbiomes significantly converged toward donor profiles (Figure 6C, *p* = 0.0317), while non-responders remained phylogenetically distinct (Figure 6 6D, *p* = 0.421). This convergence strongly predicted clinical improvement.

## Discussion

This pilot randomized controlled trial provides clinical evidence that fecal microbiota transplantation (FMT) can restore gut microbiome diversity in critically ill sepsis patients following broad-spectrum antibiotic treatment, demonstrating potential advantages over probiotic intervention. Our findings reveal that FMT preserved microbial alpha diversity against antibiotic-driven depletion, significantly increasing species richness compared to probiotic group. Beyond diversity metrics, FMT induced substantive taxonomic restructuring characterized by enrichment of beneficial *Bacteroidetes* (particularly genus *Bacteroides*) and suppression of opportunistic pathogens like *Enterobacteriaceae*. In addition, BugBase analysis suggested a potential decrease in the predicted pathogenic potential of the gut microbiota in the FMT group. These microbial changes coincided with a descriptive trend toward lower 28-day mortality (0% in FMT recipients versus 20–25% in the other groups; exploratory only). However, given the small sample size and limited statistical power, these findings should be interpreted cautiously especially clinical efficacy.

The gut microbiome and sepsis share a complex bidirectional relationship, wherein dysbiosis exacerbates systemic inflammation while sepsis further disrupts gut ecology. Current microbiome-modulating strategies include: probiotics, prebiotics, and FMT. Crucially, a balanced microbiome bolsters anti-pathogen immunity, whereas its disruption increases sepsis susceptibility and mortality. In previous studies on the gut microbiota in sepsis, Chen et al. effectively reduced mortality by prophylactically using probiotics in a mouse model [22]. More recently, a study conducted on a model of septic mice specifically demonstrated that after the onset of sepsis, there was an appearance of opportunistic gut pathogens such as *Staphylococcaceae* and *Enterococcaceae* and a disappearance of beneficial *Prevotellaceae* [23]. However, in a recent randomized controlled trial, researchers evaluated the role of prophylactic synbiotics in mechanically ventilated sepsis patients and found no significant differences between groups in the incidence of enteritis, VAP, bacteremia, or mortality [24]. This finding resonates with our study. In our study, the Probiotics group exhibited reduced diversity and increased Enterobacter abundance at 2 weeks despite probiotic administration. These were most likely attributable to the continued use of broad-spectrum antibiotics during the intervention. Antibiotics exert strong community-wide suppressive effects that substantially reduce gut microbial diversity and lead to insufficient colonization of probiotics [25]. Besides, previous studies have shown that antibiotic exposure promotes the expansion of *Enterobacteriaceae* by disrupting colonization resistance, not only through the depletion of competing commensals but also by profoundly altering the gut metabolic environment [26]. Although the administered *Bifidobacterium-Lactobacillus-Enterococcus* formulation contains viable beneficial strains, prior studies have demonstrated that probiotics cannot fully mitigate antibiotic-induced loss of diversity, nor reliably prevent the overgrowth of opportunistic taxa during antibiotic exposure [27]. These findings underscore that single probiotic or prebiotic interventions have limited capacity to counterbalance antibiotic-driven dysbiosis in critically ill patients treated with broad-spectrum antibiotics. It should be noted that the efficacy of probiotic interventions may vary substantially depending on formulation, timing, and clinical context, which limits direct comparisons across studies. Given that existing studies typically have small sample sizes and lack consensus on the optimal choice of probiotic strains and dosages, we emphasize the need for larger-scale randomized controlled trials to further investigate the clinical effects of these interventions.

The superiority of FMT likely stems from its capacity to reconstitute complex, resilient microbial ecosystems rather than introducing isolated strains. Our results revealed that successful donor-derived engraftment underpinned FMT efficacy: 60% of recipients transitioned to a *Bacteroides*-dominant enterotype mirroring donor profiles, contrasting with their baseline *Parabacteroides*-dominant ecology. This restructuring correlated strongly with colonization by donor-specific *Bacteroides* strains, supporting the hypothesis that keystone taxa drive ecological recovery. Furthermore, responders exhibiting clinical improvement demonstrated significant microbiome convergence with donors (Bray-Curtis distance decreased) and positive Colonizer-to-Resident Ratios (C2R), establishing engraftment efficiency as a potential predictor of therapeutic success. Previous studies have proposed that FMT may help increase microbial diversity and community stability, which could support gut barrier function, reduce intestinal permeability, and limit the translocation of pathogens and endotoxins into the bloodstream [28]. Consistent with these observations, our study also showed a notable restoration of gut microbial diversity following FMT, despite ongoing broad-spectrum antibiotic exposure. FMT has also been reported to promote the recovery of beneficial taxa such as *Bacteroides*, which may contribute to immune modulation through metabolites [29]. In our study, we observed an increase in *Bacteroides* abundance in FMT recipients compared with the probiotic and control groups. Additionally, FMT has been suggested to reduce the relative abundance of pathogenic bacteria and partially mitigate dysbiosis-associated immune dysfunction [30]. Our findings further support this possibility, as FMT attenuated the antibiotic-driven expansion of potential pathogenic taxa, particularly *Enterobacter*.

The application of FMT in the intensive care setting is particularly urgent and promising. In early studies, Li et al. demonstrated the potential utility of FMT in restoring gut barrier function and providing adjunctive therapy by applying it to patients with sepsis induced by ulcerative colitis [31]. In 2015, Li and his team reported in detail a patient who, after undergoing vagotomy, failed to improve from infection and diarrhea with antibiotics and supportive treatment but ultimately showed significant improvement with FMT [32]. Additionally, Wei et al. observed that two patients hospitalized for stroke treatment, after experiencing septic shock and multiple organ failure, exhibited significant relief from infection and effective regulation of immune responses following the application of FMT to restore gut microbiota [33]. Similarly, Wurm et al.‘s study further confirmed that the application of FMT in patients with persistent diarrhea and SIRS response rapidly alleviated symptoms and reduced the occurrence of complications [34]. Previous reports on the use of FMT in sepsis have been largely limited to individual case descriptions, our study provides initial observations from an exploratory randomized controlled trial with small sample size. In our study, mortality was observed lower in the FMT group (0% vs. 20–25%). Although our findings offered clinical evidence that FMT may enhance treatment outcomes in sepsis by restructuring the gut microbial ecosystem. However, given the limited sample size and pilot nature of the study, these results should be interpreted with caution and cannot be considered confirmatory. Future research should continue to explore the combined application of FMT with traditional treatment methods such as antibiotics and probiotics, as well as its performance in multi-center, large-sample clinical trials to achieve optimal therapeutic effects.

This study provides preliminary insights into the potential role of FMT in modulating the gut microbiome of critically ill patients with sepsis; however, several limitations must be acknowledged to ensure appropriate interpretation of the findings. Firstly, as an exploratory single-center pilot randomized controlled trial, the primary aim was to assess safety and gut microbiome recovery rather than to establish clinical efficacy of FMT. Accordingly, this study was not statistically powered for patient-centered outcomes. The observed 28-day mortality difference (0% in FMT group vs. 20–25% in the Control and Probiotics groups, *p* = 0.297) should therefore be viewed as descriptive rather than indicative of therapeutic benefit, particularly given baseline imbalances in age, disease severity, and antibiotic exposure. Future multicenter trials with larger sample sizes required to determine these early signals. Secondly, the lack of blinding for patients and clinicians introduces potential performance bias. Moreover, no significant improvements were observed in key clinical outcomes, including 28 day-mortality, SOFA score trajectories, or ICU length of stay, which underscores the exploratory nature of this study and limits conclusions regarding patient-level benefits. Thirdly, the functional insights based on 16S rDNA sequencing were based on predictive computational tools (PICRUSt2, BugBase) rather than direct measurements, such as metabolomics (e.g. short-chain fatty acids). And the short follow-up period further restricts insight into long-term microbial stability or delayed physiological effects. Finally, the single-center design limits generalizability. Gut microbiome composition varies considerably by geography, diet, and host genetics, and ICU practice, such as antibiotic stewardship, nutritional protocols, and infection control, differ widely across institutions and countries. These contextual factors may influence microbiome dynamics and intervention responsiveness, and broader applicability of the current findings must be confirmed in diverse clinical settings. Despite these limitations, this study offers early evidence on the feasibility and safety of administering FMT to ICU patients with sepsis-an area with extremely limited randomized data. The dataset provides a valuable foundation for future mechanistic studies and adequately powered multicenter trials aimed at clarifying the clinical relevance of microbiome-targeted therapies in sepsis.

## Conclusion

In conclusion, this exploratory single-center pilot trial provides preliminary evidence that FMT may help reverse antibiotic-associated dysbiosis in critically ill patients with sepsis. Compared with probiotics, FMT was associated with greater restoration of microbial diversity, increased abundance of beneficial taxa such as *Bacteroides*, and attenuation of antibiotic-driven expansion of potential pathogens, including *Enterobacter*. These findings suggest that FMT could be a feasible microbiome-targeted approach for modulating gut ecology in sepsis.

## Supplementary Material

Supplemental Material

## Data Availability

The datasets generated and/or analyzed during the current study are available in the Sequence Read Archive (SRA), under the BioProject ID PRJNA1166732 (https://www.ncbi.nlm.nih.gov/bioproject/?term=PRJNA1166732). The supplementary tables supporting this study have been deposited in the Science Data Bank (data DOI: 10.57760/sciencedb.35235).

## Clinical trial registration

The study was also registered on ClinicalTrials.gov (registration number: NCT05578196). In ClinicalTrials.gov, we inadvertently listed the IRB application date (2022–09-04) as the “Study Start” date. In practice, patient recruitment began only after registration was completed. No participants were enrolled or intervened prior to registration. We confirm strict adherence to prospective registration principles.
