# Supplementary figures and images for: Effects of fecal microbiota transplantation and probiotics on the gut microbiome in antibiotic-treated septic patients: A pilot randomized controlled trial
Source: Virulence. 2026 May 22;17(1):2668764. doi: 10.1080/21505594.2026.2668764 (PMC13203059; doi:10.1080/21505594.2026.2668764)

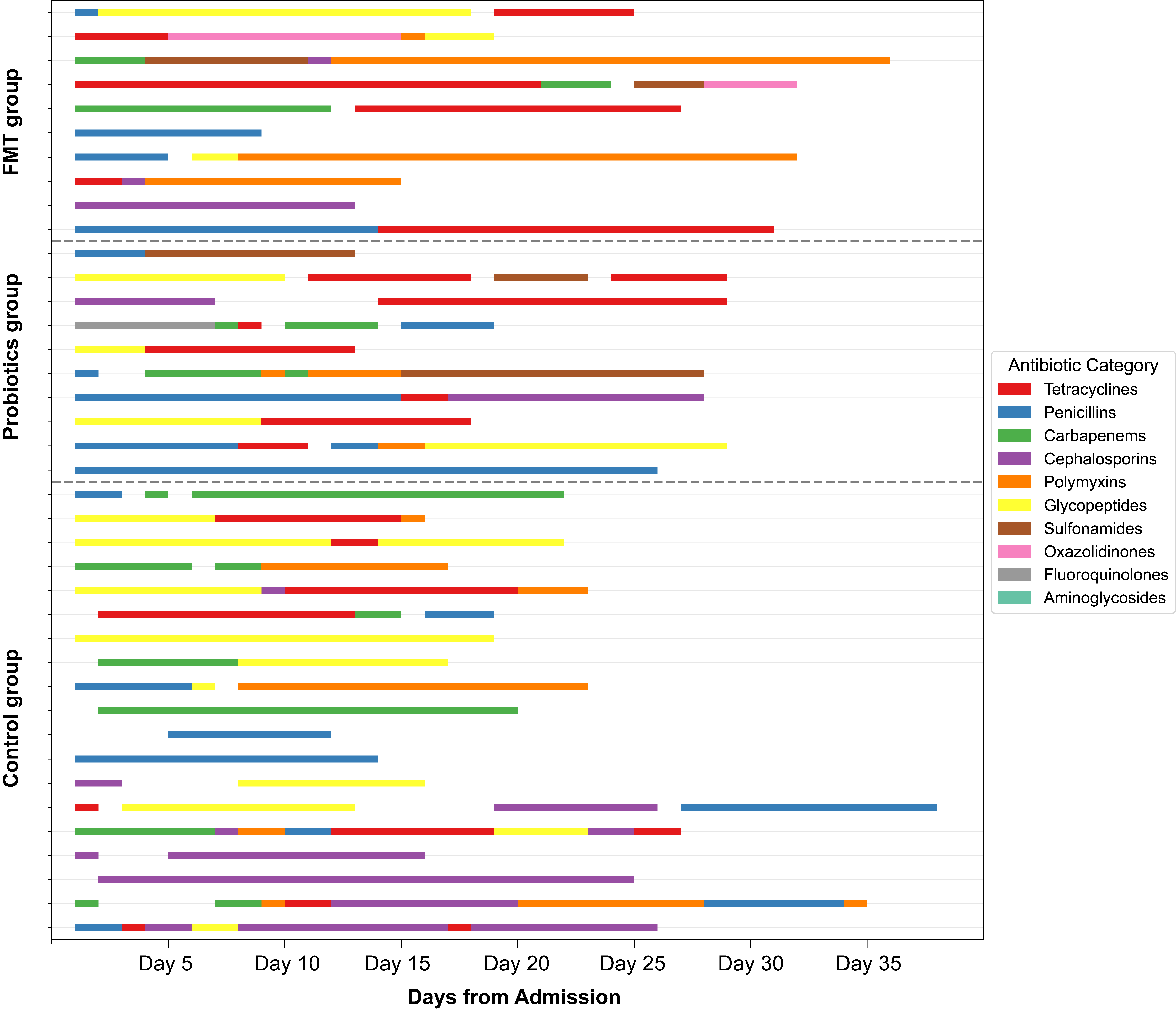

Supplement: Supplemental Material [file KVIR_A_2668764_SM3958.zip › Revised Supplementary Figure 1.tif]

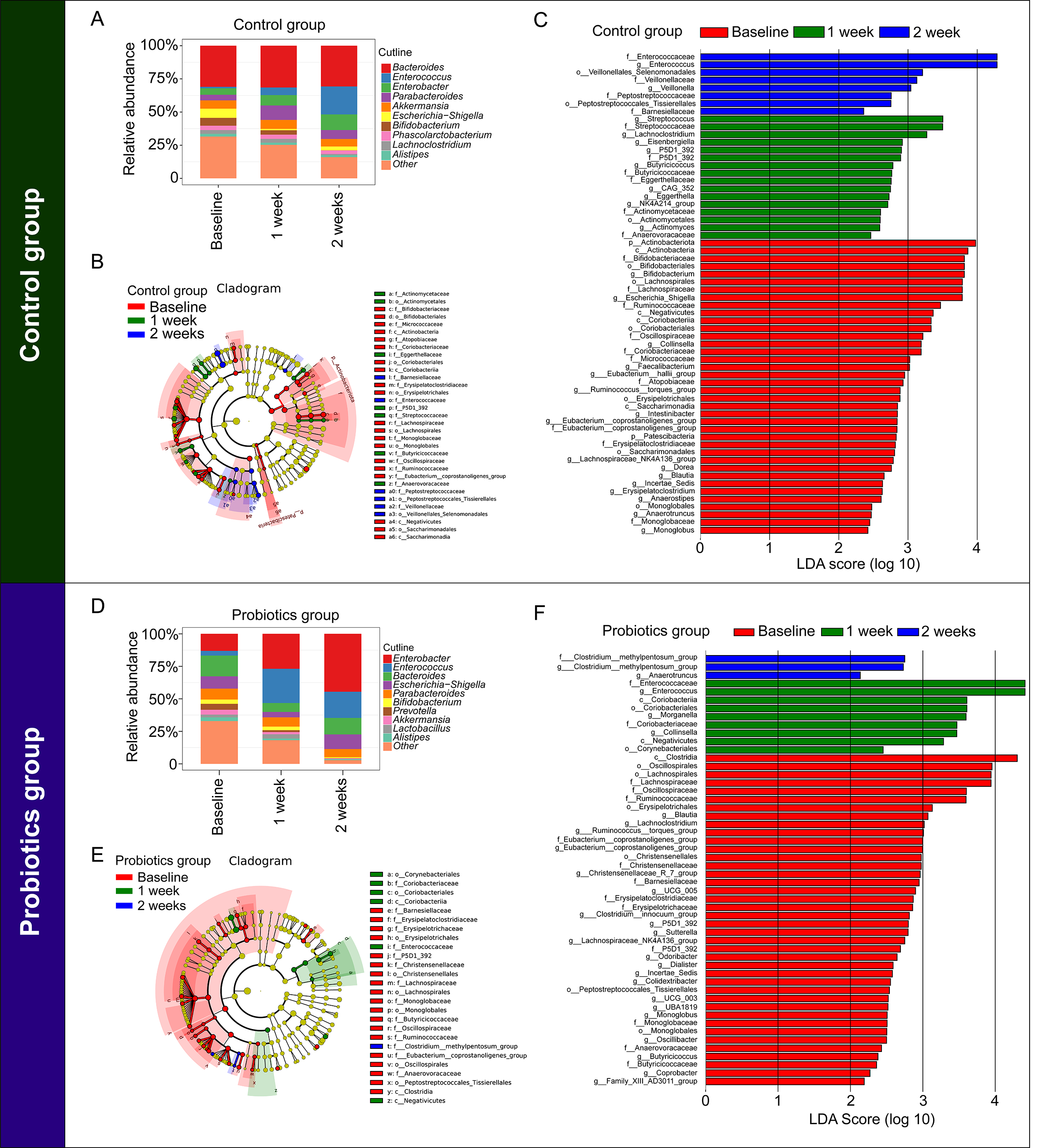

Supplement: Supplemental Material [file KVIR_A_2668764_SM3958.zip › Supplementary Figure 2.tif]

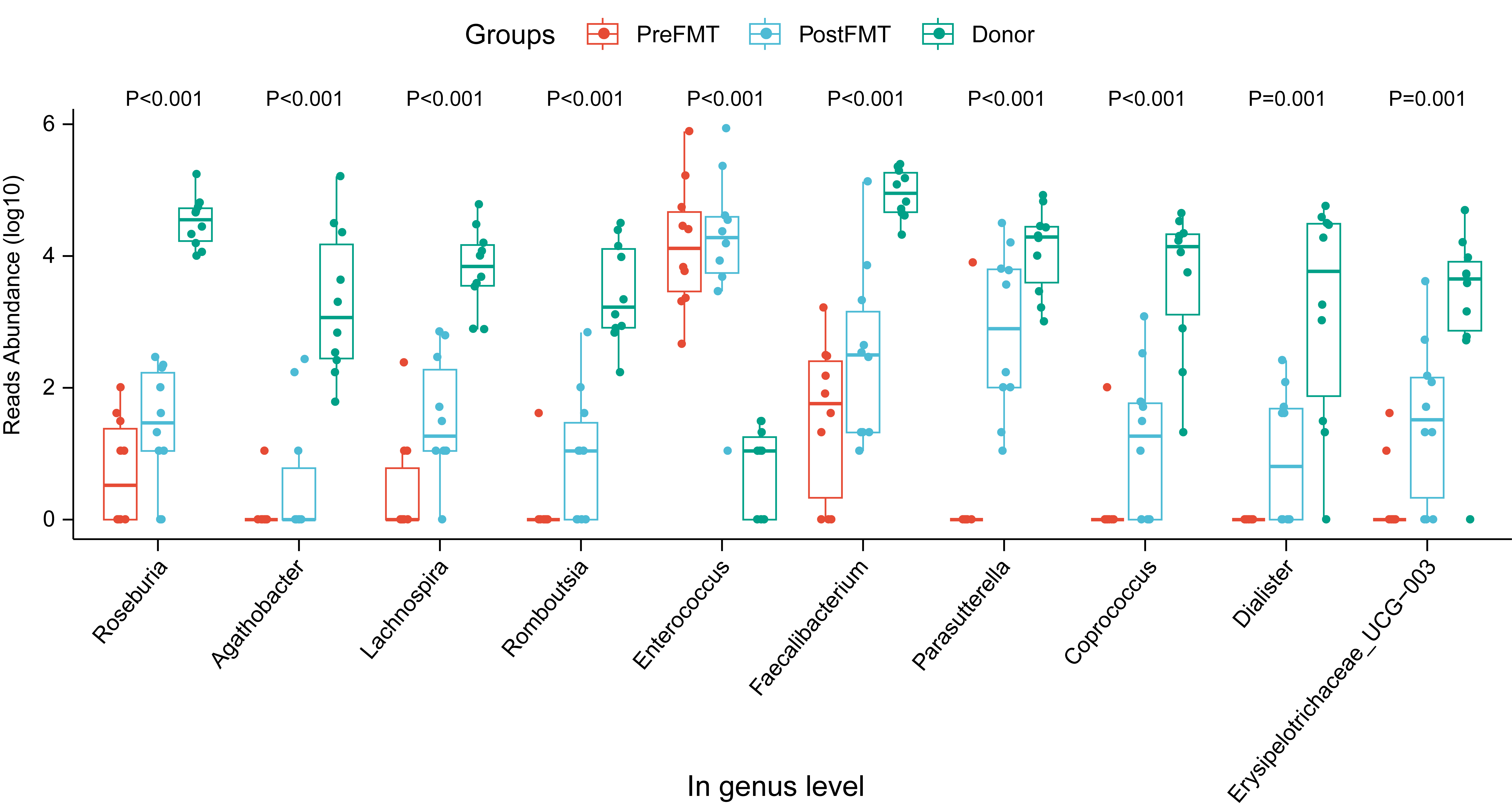

Supplement: Supplemental Material [file KVIR_A_2668764_SM3958.zip › Supplementary Figure 3.tif]
